# Dr. Robert Early

**Published:** 1884-02

**Authors:** 


### OBITUARY.

---

DR. ROBERT EARLY died at Lynchburg Virginia, December 29th, 1883, in the seventy-first year ot his age.

Dr. Early was one of the best known dental practitioners in Virginia, where, as a resident of Lynchburg, he practiced his profession for some fifty years, and enjoyed the reputation of a successful and skillful operator, being in constant practice until the year previous to his death. As a citizen he merited the respect of all who knew him, and was in every respect a conscientiuos and faithful practitioner, being highly esteemed for his integrity and and superior judgment. Dr. Early was a native of Pennsylvania, and when quite young entered the office of Dr. Ropes, of Philadelphia, as a dental student, before the days of dental colleges, of which, however, he was always a warm advocate. Removing to Lynchburg, Virginia, he established a large practice, and became one of the wealthiest dentists in the State, and attained also high social relations. As a contemporary of Prof. Chapin A. Harris, and other eminent practitioners of an early period, the name of Dr. Robert Early has long been known as that of one of the best practitioners of the old school in this country.

